# Supplementary material for: Local invertibility and sensitivity of atomic structure-feature mappings
Source: Open Res Eur. 2021 Oct 20;1:126. [Version 1] doi: 10.12688/openreseurope.14156.1 (PMC10445828; doi:10.12688/openreseurope.14156.1)
Supplement: Peer review history

# Peer review: Reviewer response for version 1

**DOI:** 10.21956/openreseurope.15263.r27875

**Published:** 2021-11-17

**Authors:** Bastiaan Braams

**Affiliations:** 1 The Dutch National Center for Mathematics and Computer Science, Centrum Wiskunde & Informatica (CWI), Amsterdam, The Netherlands

The research article under review concerns issues of uniqueness and conditioning of atomic structure feature mappings that are used in machine-learning of molecular energies and force fields. The paper elaborates on an earlier more accessible presentation by an overlapping group of authors [1] (ref. [5] in the present article) and I recommend to readers to start with [5] as an introduction to the present article. The feature maps are classified with respect to the degree of the involved correlations: one-point (\nu=1) features involving distances to the central atom only, two-point features involving distances and plane angles, three-point features also solid angles, and so on; and note that the points in this language are arbitrary points in the neighborhood of the central atom, they are not neighboring atoms. For any fixed degree \nu the correlation depends on \nu spatial coordinates and the level of discretization is another parameter; in the paper the complete basis set limit is emphasized at first and then numerical experiments are provided for specific discretizations using the potential energy surface of methane (CH4) as the primary example. Altogether the article offers an important contribution to the understanding of accuracy of machine-learned atomic force fields based on atomic neighborhood feature mappings.

 In motivating the study the authors emphasize the diversity of approaches relying on local feature mappings, and it may be useful to point out that not all machine learning methods for large molecules and materials fall in that category. In particular I highlight the SchNet approach [2] that is a forerunner of a large class of methods for machine learned force fields under the umbrella name of graph convolutional neural networks (GCNN); see the review [3] and many references therein. In the GCNN approach the identity of atoms is retained and one works with functions of the local internuclear distances. As noted also in the present paper, the matrix of internuclear distances provides a good coordinate system as the Jacobian of this feature vector is singular only when it is mandated by symmetry. Replacing each internuclear distance rij by some f(rij) where f is monotone, smooth and compactly supported, going to zero at some modest distance, one obtains a sparse edge-weighted graph with the number of edges bounded linearly in the number of atoms. This representation seems to avoid the possible ill-conditioning associated with the atomic feature mappings. (For present purposes we ignore issues of long-range electrostatics; they are a special concern in any case.) However, a comparison of methods relying on atomic feature mappings with methods from the GCNN class is outside the scope of the present work.

Is the study design appropriate and does the work have academic merit?

Yes

Is the work clearly and accurately presented and does it cite the current literature?

Yes

If applicable, is the statistical analysis and its interpretation appropriate?

Yes

Are all the source data underlying the results available to ensure full reproducibility?

Yes

Are the conclusions drawn adequately supported by the results?

Yes

Are sufficient details of methods and analysis provided to allow replication by others?

Yes

Reviewer Expertise:

Plasma Physics, Chemical Physics, Atomic and Molecular Data, Computational Science

I confirm that I have read this submission and believe that I have an appropriate level of expertise to confirm that it is of an acceptable scientific standard.

## References

[1] : Incompleteness of Atomic Structure Representations. *Phys Rev Lett* .2020;125(16) : 10.1103/PhysRevLett.125.166001 166001 10.1103/PhysRevLett.125.166001 33124874

[2] : SchNet - A deep learning architecture for molecules and materials. *J Chem Phys* .2018;148(24) : 10.1063/1.5019779 241722 10.1063/1.5019779 29960322

[3] : Benchmarking graph neural networks for materials chemistry. *npj Computational Materials* .2021;7(1) : 10.1038/s41524-021-00554-0 10.1038/s41524-021-00554-0

# Peer review: Reviewer response for version 1

**DOI:** 10.21956/openreseurope.15263.r27874

**Published:** 2021-11-16

**Authors:** Reinhard Maurer

**Affiliations:** 1 Department of Chemistry, University of Warwick, Coventry, UK; 2 Chair for Theoretical Chemistry, Technical University of Munich, Munich, Germany

The manuscript studies important mathematical properties of atomic symmetry functions that are commonly used in atomistic machine learning applications. A sensitivity analysis of common atomic structure-feature mappings is introduced to assess if such mappings retain their sensitivity to capture changes in atomic configuration in scenarios with high symmetry and when a finite basis representation is applied. The theory and analysis are described in great detail, which I have found to be highly educational.

 The authors show that symmetry-induced singularities and lack of local invertibility can limit the ability to represent energy landscapes and lead to errors, which reveals interesting variations in the quality of different features in Figure 3. Particularly the failure of the power spectrum to describe discplacements of a degenerate singularity structure is fascinating.

 The manuscript offers a comprehensive and compelling analysis of fundamental mathematical aspects, while also discussing how artefacts can materialise in realistic calculations where finite truncated basis sets are employed. It is also commendable that all underlying data and the employed software has been made available. 

 I recommend acceptance of the study. My only suggestion would be to revisit some of the figure captions to better describe individual subpanels (as for example done in Figure 1).

Is the study design appropriate and does the work have academic merit?

Yes

Is the work clearly and accurately presented and does it cite the current literature?

Yes

If applicable, is the statistical analysis and its interpretation appropriate?

Yes

Are all the source data underlying the results available to ensure full reproducibility?

Yes

Are the conclusions drawn adequately supported by the results?

Yes

Are sufficient details of methods and analysis provided to allow replication by others?

Yes

Reviewer Expertise:

electronic structure theory, materials modelling

I confirm that I have read this submission and believe that I have an appropriate level of expertise to confirm that it is of an acceptable scientific standard.
